# Recent Advances in Electron Microscopy of Carbohydrate Nanoparticles

**DOI:** 10.3389/fchem.2022.835663

**Published:** 2022-02-15

**Authors:** Yu Ogawa, Jean-Luc Putaux

**Affiliations:** Univ. Grenoble Alpes, CNRS, CERMAV, Grenoble, France

**Keywords:** electron microscopy, carbohydrates, nanoparticles, radiation sensitivity, electron tomography, electron diffraction

## Abstract

Carbohydrate nanoparticles, both naturally derived and synthetic ones, have attracted scientific and industrial attention as high-performance renewable building blocks of functional materials. Electron microscopy (EM) has played a central role in investigations of their morphology and molecular structure, although the intrinsic radiation sensitivity of carbohydrate crystals has often hindered the in-depth characterization with EM techniques. This contribution reviews the recent advances in the electron microscopy of the carbohydrate nanoparticles. In particular, we highlight the recent efforts made to understand the three-dimensional shape and structural heterogeneity of nanoparticles using low-dose electron tomography and electron diffraction techniques coupled with cryogenic transmission electron microscopy.

## Introduction

Carbohydrates, also known as sugars, glycans, and saccharides, are the most widespread and abundant biomolecules in nature. The simplest units of carbohydrates are monosaccharides. They connect to each other via glycosidic linkages to form larger molecules, namely oligo- and polysaccharides. Many carbohydrate molecules possess the ability to form nanosized particles both *in vivo* and *in vitro*. Such carbohydrate nanoparticles (NPs) represent a great potential as building blocks of functional materials in various fields from biomedical and cosmetic applications to optical and load-bearing materials (Moon et al., 2011; Ifuku and Saimoto, 2012; Gim et al., 2019; Li et al., 2021). In recent years, these biosourced NPs have attracted renewed scientific and industrial interests due to their outstanding properties, biodegradability, and renewability. In particular, cellulose NPs, also known as nanocelluloses, have been the main focus of the research on carbohydrate NPs (Isogai et al., 2011; Chauve et al., 2014; Parker et al., 2018; Delepierre et al., 2021), while other naturally-derived or recrystallized carbohydrates, such as chitin and starch nanocrystals, have been comparatively underexplored (Putaux et al., 2003; Goodrich and Winter, 2007; Fan et al., 2008a; Jin et al., 2021).

**FIGURE 1:**
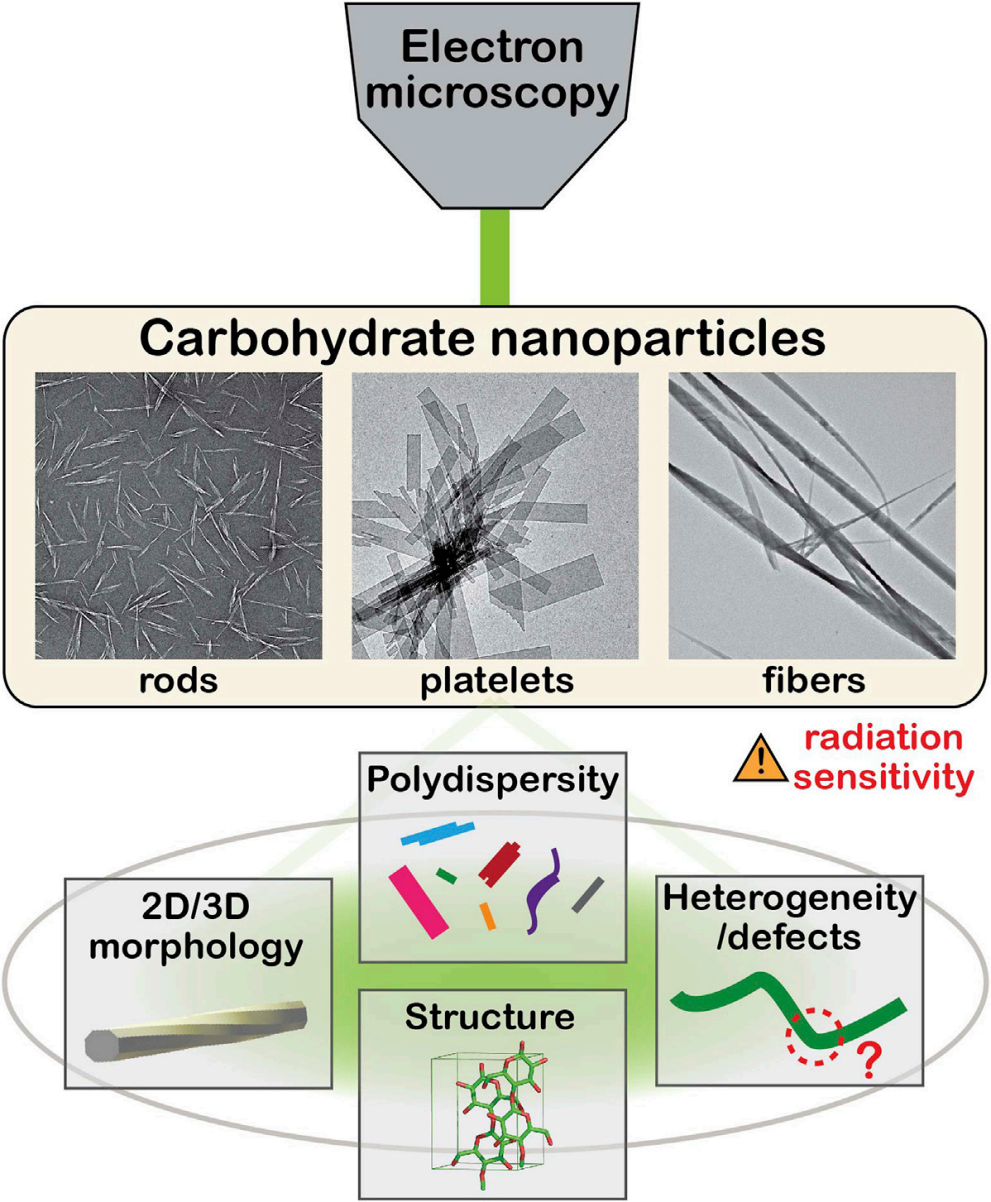
Schematic illustration of electron microscopy of carbohydrate nanoparticles.

Various microscopy techniques have been applied to these NPs to characterize their ultrastructural features (Baker et al., 1998; Hanley et al., 1992; Usov et al., 2015). Electron microscopy (EM), and especially transmission electron microscopy (TEM), are the methods of choice for the characterization of various structural and morphological aspects at the nanoscale (Figure 1), although the intrinsic radiation sensitivity and electron transparency of carbohydrate solids have often hindered the in-depth investigation by TEM. Many researchers have contributed to the EM of carbohydrate crystals in attempts to reveal their ultrastructural details, as reviewed elsewhere (Chanzy and Vuong, 1985; Ogawa et al., 2019).

The radiation sensitivity of carbohydrates, in particular cellulose, has been recognized in the early studies, as the intense electron beams used for TEM observation damaged the cellulose crystals, resulting in blurry contours and weak contrasts (Hamann, 1942; Franz et al., 1943). The global radiation damage of cellulose and chitin crystals was estimated based on the intensity decay of electron diffraction spots as a function of radiation dose. The critical dose value of these crystals is within a range of 100–1,000 e^−^/nm^2^, equal to or smaller than those of protein crystals irradiated in similar conditions (Knapek, 1982; Chanzy and Vuong, 1985; Sugiyama et al., 1985a; Saito et al., 1995). This posed a challenge in imaging well-defined morphological details of carbohydrate crystals by TEM. To overcome this obstacle, two contrasting methods were firstly applied to cellulose crystals, namely directional metal shadow-casting and negative staining with metal salts. These methods yielded well-defined images of cellulose crystals (Frey-Wyssling and Frey, 1950; Rånby, 1954; Franke and Ermen, 1969). The negative staining technique is currently the most accessible and widely used contrasting method in routine imaging of carbohydrate NPs (Ogawa and Putaux, 2019). However, the information and the resolution in the resulting images are from the contrasting agents and not from the cellulose crystals. The direct imaging of unstained and unshadowed cellulose crystals had to wait for the implementation of low-dose illumination procedures where the electron beam intensity used for imaging was significantly reduced to minimize the radiation damage. This allowed not only diffraction contrast imaging of carbohydrate crystals both in bright and dark fields (Roche and Chanzy, 1981; Näslund et al., 1988; Kim et al., 2006) but also high-resolution phase-contrast imaging in which projections of the crystal lattices were directly revealed (Sugiyama et al., 1985b; Kuga and Brown Jr, 1987; Revol et al., 1988; Helbert and Sugiyama, 1998; Imai et al., 2003; Cardoso et al., 2007; Ogawa et al., 2011). Low-dose illumination conditions have also been applied to electron diffraction experiments, allowing to understand the crystal structure at the single crystal level (Sugiyama et al., 1991). Cryogenic TEM was another crucial technological development that contributed to the EM imaging of these radiation sensitive NPs since keeping the specimen at low temperature during observation significantly reduces the radiation damage (Knapek, 1982; Henderson, 1990; Baker and Rubinstein, 2010). An extension of low-temperature methods was the observation of colloidal NPs embedded in vitreous ice after quench-freezing in liquid cryogens (cryo-TEM) (Dubochet et al., 1988).

These developments were essentially made in the 80 and 90s, during which the images and diffraction patterns were recorded mostly on photographic negatives. In the past 20 years, digital electron detectors have become widely available. The linearity, larger dynamic range, higher sensitivity and faster acquisition capability of such detectors brought significant improvement to the low-dose observation of radiation-sensitive specimens.

In this contribution, we review the recent advances of EM-based characterization of carbohydrate NPs. First, we describe different types of carbohydrate NPs, then highlight the recent efforts in characterizing their morphology and structure, using various EM methods. We have limited the scope to the description of the advances made with crystalline carbohydrate NPs. We do not discuss here other types of carbohydrate assemblies, such as nanogels and micelles.

### Variety of Carbohydrate Nanoparticles

Carbohydrate NPs occur with a large variety of shapes and size, and one or more of their dimensions lie within the range of a few to tens of nanometers. Based on their preparation methods, carbohydrate NPs can be categorized into two groups: extracted and synthetic NPs obtained via “top-down” and “bottom-up” approaches, respectively. The former group consists of traditional carbohydrate NPs prepared via chemical and/or mechanical extraction from biomass. The latter one refers to those crystallized from solutions of naturally-derived or chemically/biochemically synthesized carbohydrate molecules. The carbohydrate NPs are also classified into several groups based on their morphological features, namely nanofibers, nanocrystals, and lamellae, although the boundaries between them are not clearly defined.

Crystalline polysaccharides like cellulose and chitin generally occur *in vivo* in a nanofibrillar form, acting as structural elements in algal and higher plant cell walls, in the tunic of some sea animals and in the cuticle of arthropods (Habibi et al., 2010; Ifuku and Saimoto, 2012). Moreover, some bacteria and microalgae excrete or eject crystalline fibrils that entangle and allow the development of colonies (Fryxell et al., 1984; Ross et al., 1991). Constituted of parallel or antiparallel molecules, these slender crystallites are typically 2–50 nm wide and several micrometers long. As this morphology is controlled by the biosynthesis, that of extracted NPs depends on the parent biomass, on the processing conditions of purification and isolation (acid or enzymatic hydrolysis, mechanical homogenization, etc.) (Elazzouzi-Hafraoui et al., 2008; Reid et al., 2017), and on their drying history (Horikawa et al., 2018). In addition, the biosourced NPs are generally polydisperse due to both biosynthetic and processing factors.

Nanocelluloses are the most studied carbohydrate NPs and are extracted from plant biomass such as cotton and wood pulp as well as bacteria and tunicates (Moon et al., 2011; Thomas et al., 2018). Cellulose nanofibers (CNFs) are slender particles with a width of 3–20 nm and an aspect ratio typically larger than 100 (Abe et al., 2007; Isogai et al., 2011). Cellulose nanocrystals (CNCs) are rodlike NPs mostly obtained through acid hydrolysis of cellulosic biomass and CNFs, with a width ranging from 3 to 100 nm and an aspect ratio of up to ca. 50 (Elazzouzi-Hafraoui et al., 2008; Habibi et al., 2010). Chitin nanofibers and nanocrystals (ChNFs and ChNCs) are extracted from chitinous biomass such as crab and shrimp shells and fungi (Fan et al., 2008a, 2008b; Ifuku and Saimoto, 2012; Nawawi et al., 2020). They have a similar morphology as their cellulosic counterparts. Platelet nanocrystals can also be obtained by acid hydrolysis of native starch granules (Putaux et al., 2003).

NPs can also be prepared by *in vitro* crystallization from dilute solutions of oligo- or polysaccharides that can be either naturally-derived or chemically or enzymatically synthesized. The resulting synthetic NPs exhibit a wide variety of shapes (platelets, fibrils, rods) depending on the type of carbohydrate and crystallization conditions. Many naturally-derived linear polysaccharides, such as cellulose, chitin, amylose, xylan, or mannan, have been successfully recrystallized *in vitro* (Chanzy and Vuong, 1985; Persson et al., 1992; Hrmova et al., 2002; Heux et al., 2005; Putaux et al., 2008; Hiraishi et al., 2009; Neto et al., 2016; Meng et al., 2021). In particular, lamellar crystals similar to those of flexible synthetic polymers can be prepared. Their lateral size can reach a few micrometers but their thickness does not exceed a few tens of nanometers. The chain axis is generally perpendicular to the crystal base plane. These single crystals are well-suited for electron crystallography as their strikingly well-defined geometry is generally the expression of the symmetries of the unit cell (Chanzy and Vuong, 1985). While high molecular weight polysaccharides with a degree of polymerization (DP) above 100, can crystallize into nanosized objects, NPs with a well-defined morphology are often obtained from low molecular weight fractions, typically with a DP up to 50 with a narrow DP distribution (Chanzy and Vuong, 1985). Some carbohydrates can also form inclusion compounds when crystallized in the presence of small guest molecules (Putaux et al., 2008; Le et al., 2021). Although the *in vitro* crystallization has the potential to provide monodisperse crystals with a well-defined morphology, the resulting NPs are often as polydisperse in size as the extracted NPs. This is partly due to our limited understanding of the crystallization of carbohydrates, although its practical knowledge has been accumulated over several decades (Chanzy and Vuong, 1985).

The enzymatic synthesis is another pathway to produce synthetic carbohydrate NPs. Carbohydrate synthases and lyases (e.g., phosphorylases and hydrolases) have both been used to synthesize carbohydrates (Bracker et al., 1976; Hrmova et al., 2002; Lai-Kee-Him et al., 2002; Hiraishi et al., 2009). During the enzymatic reaction, when the DP of the synthesized molecules reaches the solubility limit, carbohydrates often spontaneously crystallize into NPs (Hrmova et al., 2002; Hiraishi et al., 2009; Kobayashi et al., 2017; Grimaud et al., 2019) or larger spherulites (Kobayashi et al., 2000; Faijes et al., 2004). There is a limited number of reports on the production of crystalline NPs based on the chemical synthesis of carbohydrates (Gim et al., 2020, 2021), even though the methodology has become well established in recent years (Seeberger, 2015). While the chemical synthesis is generally labor-demanding and time-consuming, the resulting carbohydrates have well-defined molecular structures and monodisperse DPs and therefore are ideal starting materials for controlled crystallization and NP production.

### Morphological Characterization of Carbohydrate Nanoparticles

The morphology of carbohydrate NPs has been routinely characterized using TEM, either with support from the above-mentioned contrasting methods or by direct low-dose imaging. The primary goal of these observations is to assess the particle size and their size distribution (Elazzouzi-Hafraoui et al., 2008), since the inherent polydispersity of these NPs affects their macroscopic material properties such as the rheological behavior of their suspensions (Tanaka et al., 2017; Gicquel et al., 2019). With the research community of carbohydrate NPs rapidly expanding, a few “how-to” articles have been published describing protocols for routine observations (Kaushik et al., 2015b; Ogawa and Putaux, 2019). They overview practical aspects of both sample preparation and imaging. These articles are in line with the effort to standardize analytical methods in the community, especially in nanocellulose research (Foster et al., 2018; Bushell et al., 2021). Beyond the size estimation, there are currently two challenges in the morphological characterization of carbohydrate NPs: determine the three-dimensional morphology of individual particles at high resolution and better estimate the polydispersity and aggregation state at the nano- to micrometer length scales.

#### 3D Morphology

Conventional TEM imaging provides two-dimensional images and the height information perpendicular to the observation plane is thus not accessible. It is often a problem as carbohydrate NPs are generally strongly anisometric. To obtain height information, atomic force microscopy (AFM) has been used in conjunction with EM imaging (Elazzouzi-Hafraoui et al., 2008; Yucel et al., 2021). This joint approach is practical to estimate the global size distribution. However, to better characterize the 3D morphology of the individual NPs, one has to devise a volumetric imaging method at nanometer resolution.

In recent years, electron tomography (ET) has become widely accessible to collect high-resolution 3D information on individual carbohydrate NPs. Unlike routine 2D TEM imaging, ET of carbohydrate NPs has been performed almost exclusively with unstained specimens to avoid possible artefacts in the 3D reconstruction from the staining. Tilt series acquisition of unstained, hence radiation-sensitive, particles has become possible thanks to the combination of low-dose acquisition and highly sensitive detectors sometimes combined with a zero-loss energy filter. Furthermore, the ET acquisition is frequently performed on particles embedded in vitreous ice and observed by cryo-TEM. Cryo-ET data acquisition not only reduces the radiation damage but also avoids drying artefacts that can somewhat alter the NP morphology. Ikkala and co-workers reported cryo-ET volumetric images of pristine CNCs (Majoinen et al., 2014) and CNCs grafted with polymer brushes (Malho et al., 2016) or gold particles (Majoinen et al., 2016) on their surfaces. Kaushik et al. used cryo-ET to visualize Pd patches on CNC surfaces (Kaushik et al., 2015a). More recently, Bai and co-workers applied cryo-ET to ChNCs to reveal the nanoscale fibrillar twists along their fiber axes (Bai et al., 2020).

#### Size Distribution

Carbohydrate NPs are polydisperse, both as individual objects and their aggregates. An accurate estimation of the particle size and size distribution is thus essential to correlate the nanoscale characteristics of the NPs to their macroscopic properties. One has to understand different types of contrasts of the NPs to extract the size information from the EM images. As recently reviewed elsewhere (Ogawa and Putaux, 2019), the image contrast of carbohydrate NPs contains three main contributions: amplitude contrast, diffraction (or Bragg) contrast and phase (or Fresnel) contrast. The amplitude contrast of carbohydrate NPs is generally weak as they are composed of light elements that scatter and attenuate electrons much less than heavy elements. However, this can be compensated by a strong diffraction contrast that occurs for specific orientations of crystalline NPs with respect to the incident electron beam. While it is informative in the direct imaging of unstained carbohydrate NPs, diffraction contrast is rapidly lost due to beam damage. Fresnel contrast results from sharp differences in scattering properties between adjacent regions of the specimen or between the particle surface and vacuum. This generates interference fringes at the edge of the specimen regions, whose amplitude and distribution depend on the focusing condition of the objective lens (Watt, 1997). Large under- or overfocus values result in a higher contrast but also in blurrier contours and a loss of the fine details. As this effect has a significant influence on the size estimation of NPs, one must balance the opposing requirements of contrast and structural details by conveniently adjusting the objective lens defocus.

The aggregation is commonly observed even after extensive mechanical or ultrasound homogenization. In the case of the extracted NPs, the extent of aggregation significantly varies from thin bundles of a few individual NPs with a width of several nanometers to large micron-sized agglomerates. Such polydispersity is pronounced for the extracted nanofibers, CNFs and ChNFs, as they are often insufficiently fibrillated by mechanical treatments (Mattos et al., 2019). The size heterogeneity spanning over several orders of magnitude causes a bias in the determination of size distributions since the larger aggregates are often discarded in the measurement. This is partly due to the small mesh size of TEM grids that limits the field of view. To obtain non-biased size distributions of polydisperse carbohydrate NPs, one needs to have imaging capability over lengthscales from nanometer to tens of micrometers (Kelly et al., 2021), with the help of automated image analysis procedures for statistically significant populations (Campano et al., 2021; Yucel et al., 2021). To assess the size distribution of NPs larger than a few micrometers, scanning electron microscopy (SEM) is more adapted, thanks to its wider view field compared to that of TEM and AFM. The bottleneck of SEM observation of these NPs is the limited resolution and low contrast at high magnification compared to those of TEM. Mattos et al. recently proposed “negative-contrast” SEM imaging to estimate the dimensions of fibrillar aggregates of CNFs (Mattos et al., 2019). This SEM contrasting method, based on the deposition of non-conductive CNFs on a conductive surface, is capable of imaging NPs with high contrast over lengthscales from tens of nanometers to tens of micrometers.

### Electron Diffraction Characterization of Carbohydrate Nanoparticles

Electron diffraction (ED) is a powerful tool to retrieve local structural information at the nanoscale and to correlate the molecular structure to the external morphology of crystalline NPs. Selected area ED has successfully been used when the particles were large enough in the observation plane, which was the case for lamellar crystals (Chanzy and Vuong, 1985; Putaux et al., 2011; Le et al., 2021) or fibrillar bundles (Roche and Chanzy, 1981; Saito et al., 1995). Rich patterns were recorded as the information was averaged over a large number of unit cells. However, the radiation-sensitive nature of carbohydrates hindered researchers from collecting more local information from single nanosized crystallites. In the early years of EM investigation of cellulose crystals, ED was only performed on large fibrous aggregates, resulting in fiber diffraction patterns (Preston and Ripley, 1954). In their seminal work in 1991, Sugiyama used a focused electron beam to identify two crystalline allomorphs of native cellulose, namely cellulose Iα and Iβ, coexisting along single crystallites (Sugiyama et al., 1991). This so-called nanobeam electron diffraction (NBED) method has since been applied to different carbohydrate NPs mostly for crystallographic investigations (Imai and Sugiyama, 1998; Ogawa et al., 2011). For instance, the molecular directionality of bacterial CNFs during biosynthesis (Koyama et al., 1997), of CNCs of different allomorphs (Kim et al., 2006) and of ChNCs (Hult et al., 2005) was determined by NBED combined with specific particle staining procedures.

The NBED method is useful not only for crystallographic investigations but also as a probe of the nanoscale structural heterogeneity of the NPs. Recently, two research groups used this method to revisit the fibrillar twist of nanocelluloses (Ogawa, 2019; Willhammar et al., 2021). While the twist of carbohydrate NPs has been frequently observed using various microscopy techniques, the exact twist geometry has hardly been correlated with the internal molecular packing until recently. This is partly due to the heterogeneity of the particle morphology that makes the elucidation of twist geometry challenging solely from the morphological analysis. By using NBED method, these recent studies exploited the local crystallographic information to determine the exact crystal rotation along the twisting NPs. On the one hand, cryo-TEM was combined with sequential NBED acquisition along CNFs to quantify the crystal twist along their fiber axis (Ogawa 2019, 2021). The analysis of the series of NBED patterns provided an insight into the susceptibility of the crystal twist of CNFs to the environment: the regular twist observed in aqueous suspension is partly altered or completely suppressed by the external forces such as capillary forces during the drying process and mechanical constraints in plant cell walls. On the other hand, the local crystallinity at the sharp twist regions periodically appearing along CNFs dried on a flat substrate was probed by scanning electron diffraction with a 5-nm spot size (Willhammar et al., 2021). The ED patterns indicated that the crystalline packing of cellulose molecules was maintained even in the largely twisted regions of the nanofibers. A similar NBED analysis was applied to investigate the supramolecular organization in twisted fibrillar crystallites of synthetic glucose dimers (Gim et al., 2020).

### Perspectives

The recent advances in EM have provided a better understanding of the nanoscale morphology, polydispersity, and local structural heterogeneity of several carbohydrate NPs. The technological and methodological developments in ET and ED combined with cryo-TEM have extended the capability of EM for collecting useful information from the radiation-sensitive particles. While these developments have been made mostly with nanocellulose specimens, the methods are readily applicable to other carbohydrate NPs that are relatively underinvestigated. A better understanding of the ultrastructural aspects of these particles will stimulate the research and optimize their use in functional materials.

With such advanced EM techniques, one may envisage the structural investigation of the defects such as kinks along nanofibers or structural mosaicity in larger platelet crystals. These disordered regions are underexplored by the experimental means due to the lack of suitable analytical tools but are relevant to the material properties. Their EM characterization will help bridge the gap between the ultimate properties of the single NPs and those of macroscopic materials. High-resolution lattice imaging of the carbohydrate NPs of small crystallite size is another area where further development is expected thanks to the sensitive electron detectors. So far, lattice imaging of carbohydrate crystals has been done only with relatively large crystallites with a crystallite size larger than ca. 10 nm. While its application to such smaller crystallites would be significantly more challenging, it will provide invaluable information on the morphogenesis of these NPs both *in vivo* and *in vitro*.

## Abbreviations

NP, nanoparticle; EM, electron microscopy; TEM, transmission electron microscopy; Cryo-TEM, cryo-transmission electron microscopy; ED, electron diffraction; NBED, nanobeam electron diffraction; ET, electron tomography; SEM, scanning electron microscopy; AFM, atomic force microscopy; CNF, cellulose nanofibril; CNC, cellulose nanocrystal; ChNF, chitin nanofibril; ChNC, chitin nanocrystal.
